# Histone Deacetylase 3 Inhibition Decreases Cerebral Edema and Protects the Blood–Brain Barrier After Stroke

**DOI:** 10.1007/s12035-022-03083-z

**Published:** 2022-10-18

**Authors:** Hui Lu, Ryan Ashiqueali, Chin I Lin, Aashlesha Walchale, Victoria Clendaniel, Rudy Matheson, Marc Fisher, Eng H. Lo, Magdy Selim, Amjad Shehadah

**Affiliations:** 1 Department of Neurology, Beth Israel Deaconess Medical Center, Stroke and Cerebrovascular Diseases Division, Harvard Medical School, 330 Brookline Avenue, Boston, MA 02215 USA; 2 Xuan Wu Hospital/Capital Medical University, Xicheng District, Beijing, 100053 China; 3 Department of Neurology, Beth Israel Deaconess Medical Center, Boston, MA 02215 USA; 4 Neuroprotection Research Laboratory, Departments of Radiology and Neurology, Massachusetts General Hospital and Harvard Medical School, Charlestown, MA 02129 USA

**Keywords:** Histone deacetylase 3 (HDAC3), RGFP966, ZO-1, Claudin-5, Inflammation, MMP-9, Microglia, Astrocytes, Aquaporin-4

## Abstract

We have previously shown that selective inhibition of histone deacetylase 3 (HDAC3) decreases infarct volume and improves long-term functional outcomes after stroke. In this study, we examined the effects of HDAC3 inhibition on cerebral edema and blood–brain barrier (BBB) leakage and explored its underlying mechanisms. Adult male Wistar rats were subjected to 2-h middle cerebral artery occlusion (MCAO) and randomly treated i.p. with either vehicle or a selective HDAC3 inhibitor (RGFP966) at 2 and 24 h after stroke. Modified neurological severity scores (mNSS) were calculated at 2 h, 1 day, and 3 days. H&E, Evans blue dye (EBD) assay, and fluorescein isothiocyanate (FITC)-dextran were employed to assess cerebral edema and BBB leakage. Western blot for matrix metalloproteinase-9 (MMP9), MMP-9 zymography, and immunostaining for HDAC3, GFAP, Iba-1, albumin, aquaporin-4, claudin-5, ZO-1, and NF-kB were performed. Early RGFP966 administration decreased cerebral edema (*p* = 0.002) and BBB leakage, as measured by EBD assay, FITC-dextran, and albumin extravasation (*p* < 0.01). RGFP966 significantly increased tight junction proteins (claudin-5 and ZO-1) in the peri-infarct area. RGFP966 also significantly decreased HDAC3 in GFAP + astrocytes, which correlated with better mNSS (*r* = 0.67, *p* = 0.03) and decreased cerebral edema (*r* = 0.64, *p* = 0.04). RGFP966 decreased aquaporin-4 in GFAP + astrocytes (*p* = 0.002), as well as, the inflammatory markers Iba-1, NF-kB, and MMP9 in the ischemic brain (*p* < 0.05). Early HDAC3 inhibition decreases cerebral edema and BBB leakage. BBB protection by RGFP966 is mediated in part by the upregulation of tight junction proteins, downregulation of aquaporin-4 and HDAC3 in astrocytes, and decreased neuroinflammation.

**Supplementary Information:**

The online version contains supplementary material available at 10.1007/s12035-022-03083-z.

## Introduction

Stroke is a major cause of serious disability and a leading cause of death in the USA [1]. Ischemic stroke triggers complex pathophysiological events that result in brain damage and cerebral edema [2]. Malignant cerebral edema is one of the most devastating complications of ischemic stroke, with a mortality rate of up to 80% in untreated patients [3]. Decompressive hemicraniectomy is a lifesaving procedure for patients with malignant brain edema [4]. However, hemicraniectomy carries significant morbidity and mortality and is often considered late in the clinical course of brain edema, as a last resort. Thus, there is an unmet need for new treatment options that can help prevent or slow the development of malignant edema after stroke [5].

The cascades of events after acute ischemic stroke that lead to increased blood–brain barrier (BBB) permeability and cerebral edema formation are diverse [6, 7] and include disruption of tight junction(TJ) proteins [8], upregulation of glial water channel aquaporin-4 (AQP4) [9], and an inflammatory response involving activation of microglia [10] and upregulation of matrix metalloproteinase-9 (MMP-9)[11] and NF-kB [12].

Emerging evidence suggests that epigenetic regulation plays an important role in cerebral edema [13]. Posttranslational modifications to chromatin (e.g., acetylation) have profound effects on gene expression and thus provide a multifaceted mechanism for regulation. Histone deacetylases (HDACs) are a superfamily of chromatin-modifying enzymes that silence transcription through the modification of histones by the removal of acetyl groups [14, 15]. Stroke induces a global reduction in acetylation levels of histones H3 [16–20] and H4 [21] in the ischemic brain. Histone deacetylase 3 (HDAC3) is the most highly expressed class I HDAC in the brain [22, 23]. HDAC3 is expressed in neurons [24–26], astrocytes [24, 26], microglia [27, 28], and oligodendrocytes [29]. HDAC3 is primarily localized to the nucleus, but it can shuttle between the nucleus and cytoplasm [26]. Baltan et al. showed that the subcellular localization of HDAC3 is mixed [24]. In neurons, the expression was mainly nuclear but also in the proximal axons [24], and in astrocytes, HDAC3 was localized to both the nuclei and the cytoplasm [24].

Ischemic stroke induces upregulation of HDAC3 in the peri-infarct area [24, 30]. HDAC3 expression is increased in the brain of aging [31] and diabetic mice [32]. Several laboratories have reported that selective inhibition of HDAC3 after cerebral ischemia is beneficial and represents a novel therapeutic target [27, 33–35]. We have previously shown that selective inhibition of HDAC3 decreases infarct volume and improves long-term functional outcomes after stroke [25]. Liao et al. [27] showed that HDAC3 inhibition ameliorates ischemic reperfusion injury via microglial regulation, while Zhang et al. [35] showed that HDAC3 inhibition ameliorates ischemic brain injury by regulating the inflammasome. Inhibition of HDAC3 has also been shown to reduce ischemic reperfusion injury [33] and BBB permeability in diabetic mice [32]. In this study, we examined the effects of HDAC3 inhibition on cerebral edema and BBB leakage and explored its underlying mechanisms.

## Materials and Methods

All experiments were strictly conducted in accordance with the Institutional Animal Care and Use Committee (IACUC) of Beth Israel Deaconess Medical Center.

### Drug Properties, Dose, and Timing of Administration

RGFP966 (a selective HDAC3 inhibitor) was purchased from Biorbyt (Cambridge, UK). RGFP966 is an N-(o-aminophenyl)carboxamide HDAC inhibitor [36–38]. With systemic administration, the distribution of RGFP966 to the CNS is relatively efficient, with a brain: plasma ratio of 0.45 [39]. At the dose of 10 mg/kg, RGFP966 reaches proper concentration in the brain and it acts as a selective inhibitor for HDAC3 in vivo [26, 39]. Our previous study showed that RGFP966 at the dose of 10 mg/kg crosses the BBB and significantly increases histone 3 acetylation (AcH3) in the ipsilateral hemisphere compared to vehicle control [25].

Our unpublished data showed that HDAC3 level significantly increases in the brain as early as 3 h after the induction of MCAO. Since the maximal brain concentration of the drug in the brain is reached within 30 min to 1 h after administration [39], we administered the first dose at 2 h, to ensure proper inhibition of HDAC3 before its level starts to increase at 3 h after stroke.

### Animal Middle Cerebral Artery Occlusion (MCAO) Model

As previously described [40], adult male Wistar rats (270–300 g, 2–3 months) were anesthetized with isoflurane, administered via a precision vaporizer in oxygen (3.5–5% for induction, followed by 1.5% for maintenance). Body temperature was maintained at 37 ± 0.5 °C throughout the surgical procedure using a heating pad and a feedback-regulated water heating system. A 4–0 nylon suture with its tip rounded by heating near a flame was inserted into the external carotid artery (ECA) through a small puncture. The nylon suture, whose length was determined by the animal’s weight, was gently advanced from the ECA into the lumen of the internal carotid artery (ICA) until the suture blocked the origin of the middle cerebral artery (MCA). The nylon suture was retained inside the ICA for 2 h, and the neck incision was closed. The animals were moved to their cage to awaken. Animals were re-anesthetized with isoflurane after 2 h, and restoration of blood flow was performed by the withdrawal of the suture until the tip cleared the lumen of the ECA. The incision was then closed.

### Pain Management Before and After MCAO

Buprenorphine SR is a sustained-release formulation of buprenorphine that provides up to 72 h of analgesia. Buprenorphine SR 1.2 mg/kg was administrated subcutaneously (SQ) to all animals before surgery. After MCAO surgery, all animals were monitored twice a day for any signs of pain or discomfort until the time of sacrifice, and Buprenorphine SR 1.2 mg/kg SQ was administrated once for 72 h if needed. Supportive care, including DietGel 76A, saline SQ, and heated pad, was also provided.

### Experimental Groups

To examine the effects of HDAC3 inhibition on cerebral edema and BBB leakage, adult male Wistar rats were subjected to 2-h MCAO, and randomly selected animals were treated i.p. with either vehicle (1% Tween 80) or a selective HDAC3 inhibitor (RGFP966, 10 mg/kg) at 2 and 24 h after MCAO. Before each administration, RGFP966 was freshly dissolved in 1% Tween 80. Rats were sacrificed 3 days after 2-h MCAO for histological and immunohistochemistry analysis (*n* = 6/group) and at 48 h for EBD and Western blot assays (*n* = 4/group).

### Modified Neurological Severity Score (mNSS) and Inclusion and Exclusion Criteria

mNSS is a composite of motor, sensory, balance, and reflex tests. mNSS is graded on a scale of 0 to 18 (normal score: 0; maximal deficit score: 18). One point is awarded for the inability to perform the test or for the lack of a tested reflex; thus, the higher the score, the more severe is the injury [41, 42].

mNSS was employed as a prespecified severity inclusion and exclusion criteria [43]. Specifically, if the animal’s score was mNSS 5 or more after MCAO surgery, it was included in the randomization. Animals that scored less than 5 were excluded. For each experimental animal, mNSS was performed before MCAO, at 2 h, 1 day, and 3 days after MCAO.

### Tissue Preparation for Immunohistochemistry

Animals were anesthetized with ketamine (80 mg/kg) and xylazine (13 mg/kg) via i.p. injection. The animals were then subjected to cardiac puncture and perfused with saline followed by 4% paraformaldehyde (4% PFA) via a needle inserted into the left ventricle of the heart. The brains were removed, fixed in 4% PFA overnight, and then embedded in 20% sucrose for 2 days. Using a rat brain matrix (Braintree Scientific, MA), each forebrain was cut into 2-mm-thick coronal blocks for a total of seven blocks per animal.

### Quantification of Cerebral Edema

A series of 10-µm-thick sections were cut from each block and stained with hematoxylin and eosin (H&E) for calculation of the cerebral edema for each group, as previously described [44, 45]. Each H&E–stained coronal section was digitized under the 2.5 × objective of Celestron Digital Microscope Pro. and analyzed using NIH ImageJ software. Cerebral edema was calculated by subtracting the volume (mm^3^) of the contralateral hemisphere from the ipsilateral hemisphere [44, 45].

### Immunohistochemistry

A series of coronal Sects. (10 µm thick) were obtained from the center of the lesion (Bregma − 1 to + 1 mm) and mounted on slides for analysis. For immunohistochemistry, the following primary antibodies were employed: anti-HDAC3 (Abcam, ab32369, 1:500), anti-GFAP (Millipore, MAB3026, 1:600), anti–Iba-1 (Novus Biologicals, NB100-1028ss, 1:500), anti-albumin (Invitrogen, MAB1455, 1:400), anti-AQP4 (Milipore Sigma, AB3594, 1:200), anti–claudin-5 (Santa Cruz, sc-374221, 1:400), and anti-ZO-1 (Invitrogen, 61–7300, 1:400). Negative controls were performed by omitting the primary antibody. Nuclei were visualized with 4′,6-diamidino-2-phenylindole (DAPI).

### Measurement of Fluorescein Isothiocyanate (FITC)-Dextran Extravasation

To examine the paracellular permeability of the BBB and the degree of disruption, ischemic rats were subjected to intravenous administration of high molecular weight plasma tracer-FITC-dextran [46, 47] (70 kDa molecular weight, 50 mg/rat, Sigma) 48 h after MCAO and were euthanized 15 min after FITC-dextran injection (6/group) [47]. Coronal sections from the center of the ischemic lesion (Bregma − 1 mm to + 1 mm) were obtained [48].

### Image Acquisition and Quantitation

For quantitative measurements, immunostained coronal sections and FITC-dextran were digitized using a 20–40 × objective epifluorescence (Nikon Eclipse E600) microscope. Four–six fields of view were acquired from the peri-infarct cortex, and the total number of immunoreactive cells was counted using NIH ImageJ software. The total number of positive cells per mm^2^ area is presented. The area of FITC-dextran extravasation was measured and presented as a percentage positive area of FITC-dextran [47, 48].

### Evans Blue Dye (EBD) Assay

As previously described [49], 2% solution of EBD (Sigma, St. Louis, MO) was prepared in 0.9% saline and injected to the tail vein. After 2–4 h of EBD injection, the rats were sacrificed and perfused transcardially with 0.9% of saline. The cerebral hemispheres were separated and homogenized in N,N-dimethylformamide (Sigma, MO) and then incubated for 72 h in a water bath at 55 °C. The samples were centrifuged at 1500 g for 20 min. The extracted EBD in the supernatant was quantified by absorbance at 620 nm. Results were expressed as micrograms of Evans blue per gram of brain hemisphere by comparison with solution standards and reported as fold change compared to the contralateral hemisphere.

### Western Blot

Animals were sacrificed and brain tissues were harvested and then snap-frozen in liquid nitrogen and stored at – 80 °C. Tissues were thawed, washed in ice-cold PBS, and lysed in RIPA buffer containing protease inhibitors (Sigma). Samples were then sonicated, incubated on ice for 30 min, and centrifuged at 10,000 g for 20 min at 4 °C. Protein concentration in the supernatant was determined by Pierce BCA Protein Assay Kit (Life Technologies). Equal amounts of protein (20 µg) were combined with loading buffer, boiled for 5 min, and loaded onto 4 to 20% precast polyacrylamide gel (Bio-Rad Laboratories). Separated proteins were transferred onto nitrocellulose membranes, blocked with casein-based blocking reagent (I-Block, Life Technologies) for 60 min at room temperature, and then incubated overnight at 4 °C with the following primary antibodies: MMP-9 (Cell signaling, #13,667, 1:1000) and acetyl NF-kB( cell signaling, S2S3J, 1:1000). Secondary antibodies used were HRP-linked specific for rabbit (1:2000, cell signaling) and mouse (1:2000, Cell Signaling). After incubation, membranes were washed with PBS-T and exposed to the appropriate horseradish peroxidase-linked secondary antibody. Blots were developed with Clarity Western ECL Substrate (Bio-Rad Laboratories) and detected using a BioRad ChemiDoc Touch Imaging System (BioRad Laboratories). Data were analyzed using ImageJ software. The total abundance of target protein was normalized to appropriate endogenous control and reported as fold change.

### MMP-9 Zymography

The activity of MMP-9 was measured using MMP-9 zymography [18, 50]. Human recombinant MMP-9 (Abcam, ab285785, 20 ng) was used as a standard positive control [51]. Protein samples were prepared similarly as in Western blot [50]. As described by Wang et al. [18], an equal amount of protein from each sample was incubated for 1 h with gelatin-Sepharose 4B (71,709,400 AG, GE Healthcare, Marlborough, MA, USA) with constant shaking. The pellets were washed with a working buffer (lysis buffer without Triton X-100) and resuspended in 100 mL of elution buffer (working buffer with 10% dimethylsulfoxide) for 30 min and then centrifuged [18]. The samples were loaded on 10% Zymogram Gelatin Gels (Invitrogen, Carlsbad, CA, USA). After electrophoresis, the gels were incubated in Renaturing Buffer (Invitrogen, Carlsbad, CA, USA) for 1 h at room temperature and then in developing buffer (Invitrogen) for 48 h at 37 °C. The gels were stained for 1 h in 1% Coomassie blue (Invitrogen) and then washed with distilled water for clearer background for photography [52].

### Blinded Assessment of Outcomes

All measurements (mNSS testing, cerebral edema calculation, and immunohistochemical measurements) were performed by an investigator who had no knowledge of the experimental groups and to which an animal belongs in line with STAIR criteria [43, 53].

### Statistical Analysis

Based on our published and unpublished data, the expected effect size for the independent samples *t*-test (Cohen’s *d*) for mNSS at 3 days is 2. The sample size (*n* = 6) of each group was selected based on power analysis calculation using G*Power with an alpha level of 0.05, power of 80%, and an expected large effect size of 2.

An unpaired student *t*-test was used to test differences in histological measures among the treatment groups. One-way analysis of variance (ANOVA) with post hoc Bonferroni test was used for data analysis of multiple group experiments. Repeated measures two-way ANOVAs were performed for functional tests. Spearman or Pearson correlation coefficients were calculated among the immunostaining evaluation measurements and their correlation with functional outcome and cerebral edema. Statistical significance was set at *p*-value < 0.05.

## Results

### Selective Inhibition of HDAC3 with RGFP966 Improves Functional Outcomes and Decreases Cerebral Edema and BBB Leakage

**Fig. 1:**
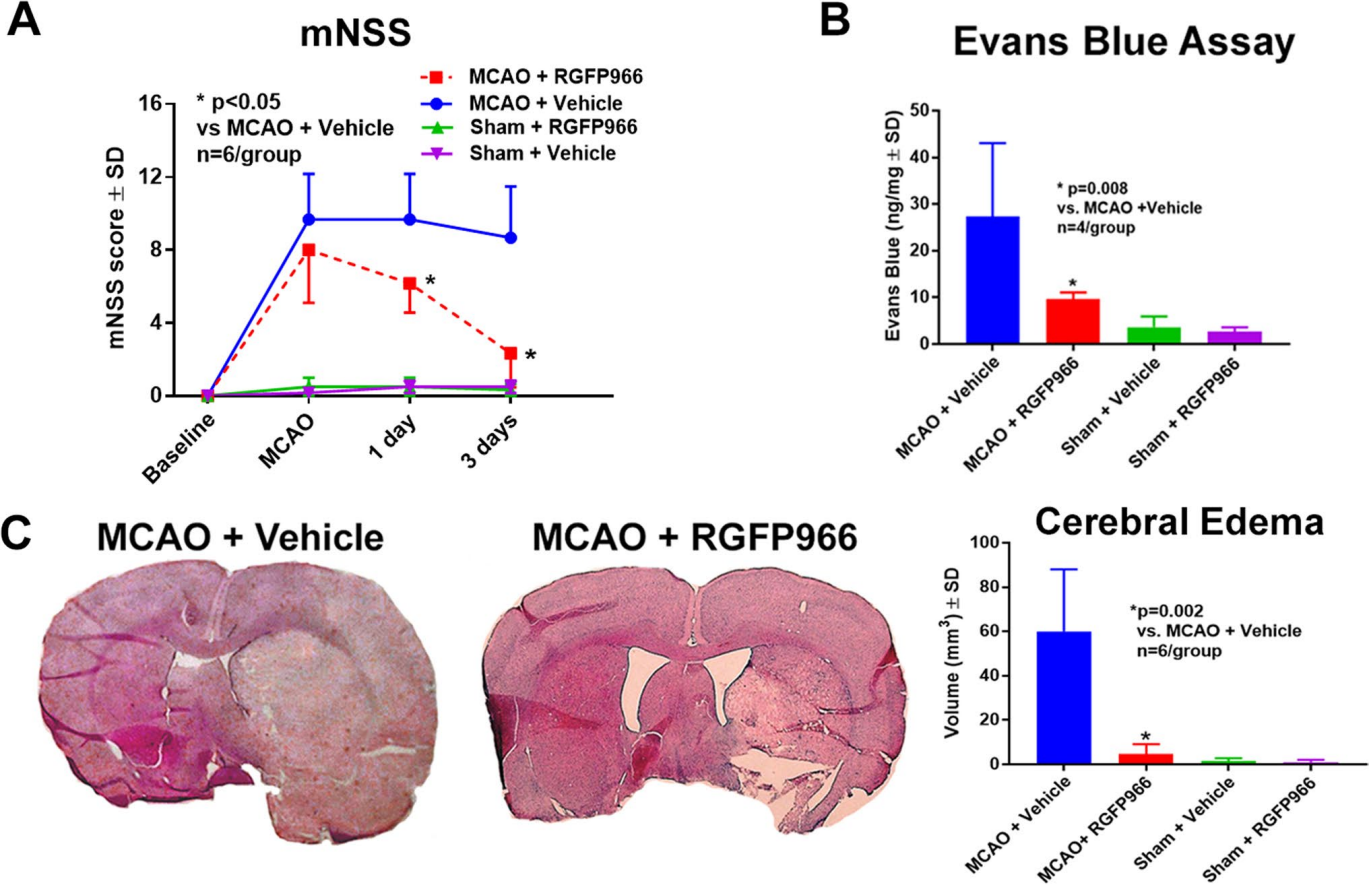
RGFP966 improves neurological outcome and decreases cerebral edema and BBB leakage. **A** RGFP966 improves neurological severity scores at 1 and 3 days after MCAO compared to the control group. **B** RGFP966 decreases BBB leakage, as measured by Evans blue dye extravasation, compared to the control group. **C** RGFP966 decreases cerebral edema, as measured by H&E, compared to the control group. SD, standard deviation

To investigate whether selective inhibition of HDAC3 improves neurological outcome, another set of rats was subjected to 2 h of MCAO, and randomly selected animals were treated intraperitoneally with two doses of either vehicle (1% Tween 80) or a selective HDAC3 inhibitor (RGFP966, 10 mg/kg) at 2 and 24 h after MCAO. Behavioral tests were performed at 2 h, 1 day, and 3 days after MCAO. Repeated measure ANOVA showed that at 1 and 3 days after MCAO, RGFP966 significantly improved mNSS compared to the control group (Fig. 1A). The mortality rates were 25% in the vehicle control group (2 died out of 8 and we had 6 animals total for analysis) and 14% in the treatment group (1 died out of 7 and we had 6 animals total for analysis).

**Fig. 2:**
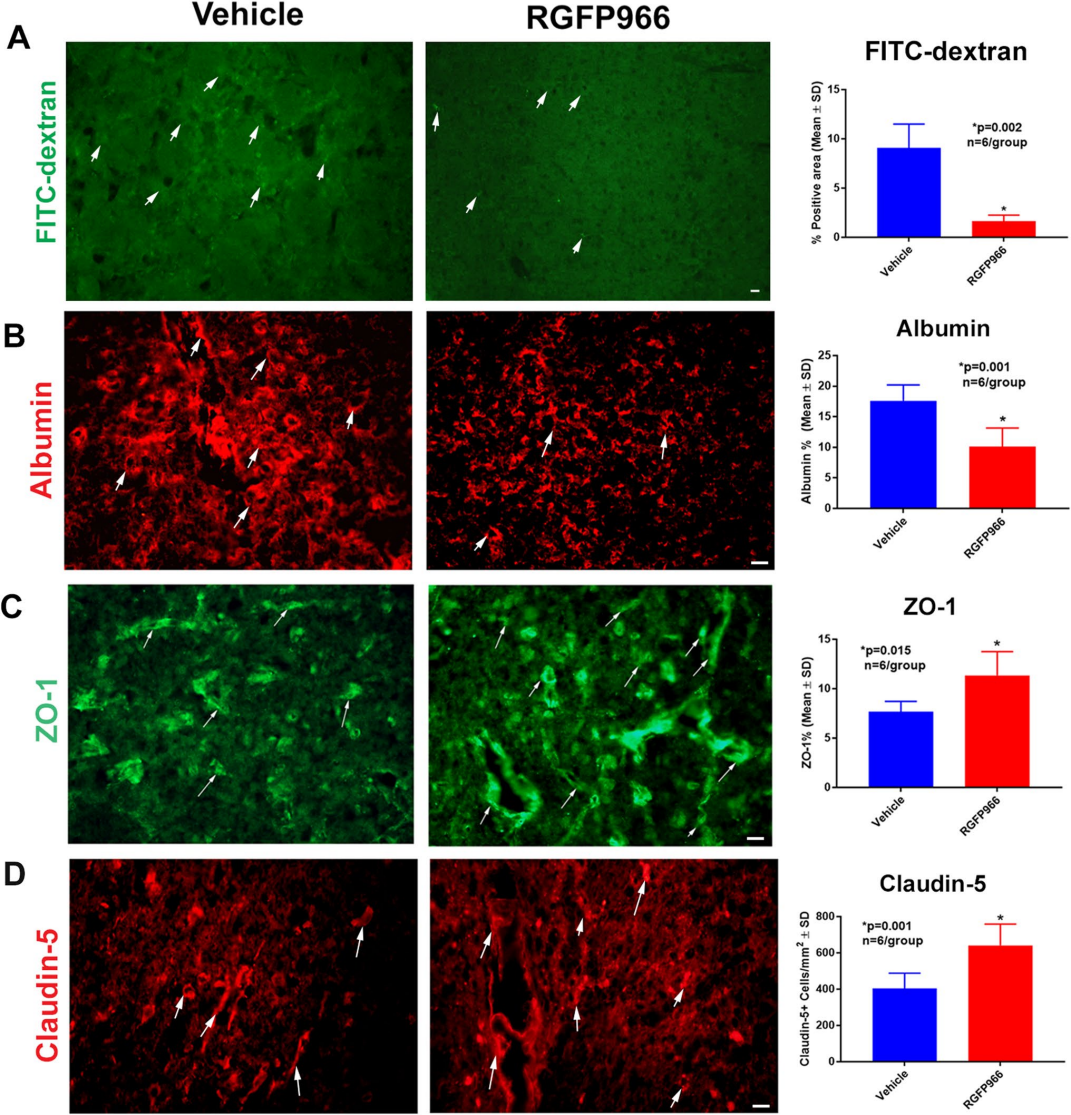
RGFP966 decreases the extravasation of FITC-dextran and albumin and increases ZO-1 and claudin-5 in the peri-infarct cortex. **A**, **B** RGFP966 significantly decreases the extravasation of FITC-dextran (**A**) and albumin (**B**) in the peri-infarct cortex compared to the control group. RGFP966 also increases the ZO-1 (**C**) and claudin-5 (**D**) in the peri-infarct cortex. SD, standard deviation

We further investigated whether RGFP966 decreases cerebral edema using H&E staining and found that RGFP966 significantly decreased cerebral edema compared to the control group (Fig. 1C). Next, we used an EBD assay to measure the degree of BBB leakage and whether selective HDAC3 inhibition decreases BBB. Our data showed that selective inhibition of HDAC3 significantly decreased the extravasation of EBD compared to the control group (Fig. 1B). To examine the paracellular permeability of the BBB and the degree of disruption, we administered FITC-dextran after MCAO, measured its extravasation in the ischemic brains, and found that RGFP966 significantly decreased the extravasation of FITC-dextran (Fig. 2A). Immunostaining also confirmed that RGFP966 decreased the extravasation of albumin (Fig. 2B).

### RGFP966 Increases Tight Junction Proteins Immunostaining in the Peri-infarct Cortex

To investigate the underlying mechanism of HDAC3 regulation of BBB leakage, we used immunohistochemistry to measure the levels of tight junction proteins in the peri-infarct cortex. Our data showed that RGFP966 increased the ZO-1 and claudin-5 (Fig. 2C, D, respectively). We performed Western blot for claudin-5 and ZO-1 and found no difference between the groups (*n* = 3/group). Our explanation for the differences between immunostaining measurements and the Western blot results is that for immunostaining, the peri-infarct area is identified under the microscope and images are carefully acquired at the border zone between the normal tissue and the infarcted area. In contrast, brain tissue for Western blot is obtained macroscopically, and as a result, it contains the infarcted area, the peri-infarct area, and normal tissue; this may be responsible for the loss of signal that we observed with immunostaining.

### RGFP966 Decreases HDAC3 and AQP4 in GFAP-Positive Cells

**Fig. 3:**
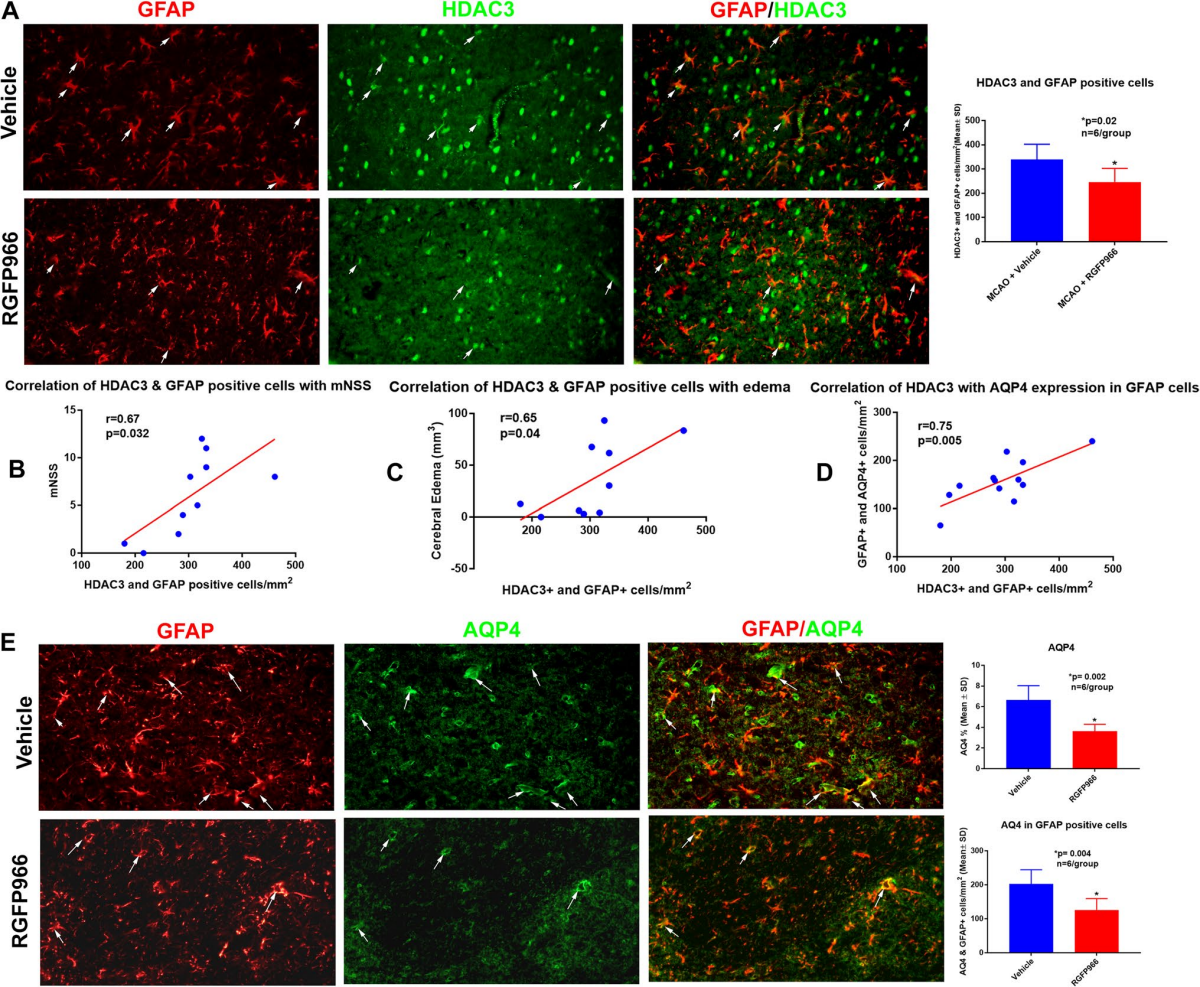
RGFP966 reduces HDAC3 and AQP4 in GFAP-positive cells. **A** RGFP966 significantly decreases HDAC3 in GFAP-positive cells. This reduction was correlated with reduced neurological severity scores (**B**) and decreased cerebral edema (**C**). **D** HDAC3 levels correlate with AQP4 in GFAP-positive cells. **E** RGFP966 decreases the total AQP4 and AQP4 in GFAP-positive cells. SD, standard deviation

Astrocytes are the most common glial cells in the brain and play a significant role in health and disease [54]. Astrocyte end feet cover more than 90% of the brain capillary [55] and form the glial limitans which is a unique characteristic of the BBB [56]. Several studies have established the critical role of astrocytes in the maintenance of the structure and function of BBB [57–59]. One of the early responses after cerebral ischemia is astrocyte swelling, which is in part due to the translocation of AQP4 to the cell surface [60]. In the brain, AQP4 is mainly expressed in the astrocytes [61] and it has been shown to facilitate cerebral edema and astrocytic swelling [62–64]. To further investigate the effect of HDAC3 on BBB, we examined whether the regulation of HDAC3 in astrocytes is associated with a reduction of AQP4 and whether these changes correlate with a reduction of cerebral edema and improved functional outcome. Our double immunohistochemistry showed that RGFP966 decreased HDAC3 in GFAP-positive cells (Fig. 3A) and also decreased the total AQP4 as well as AQP4 in GFAP-positive cells (Fig. 3E). A significant correlation was observed between the HDAC3 and AQP4 in GFAP-positive cells (Fig. 3D), and the reduction of HDAC3 in GFAP-positive cells was correlated with decreased cerebral edema (Fig. 3C) and better neurological scores (Fig. 3B).

### RGFP966 Decreases Microglia and Inflammatory Markers in the Peri-infarct Cortex

**Fig. 4:**
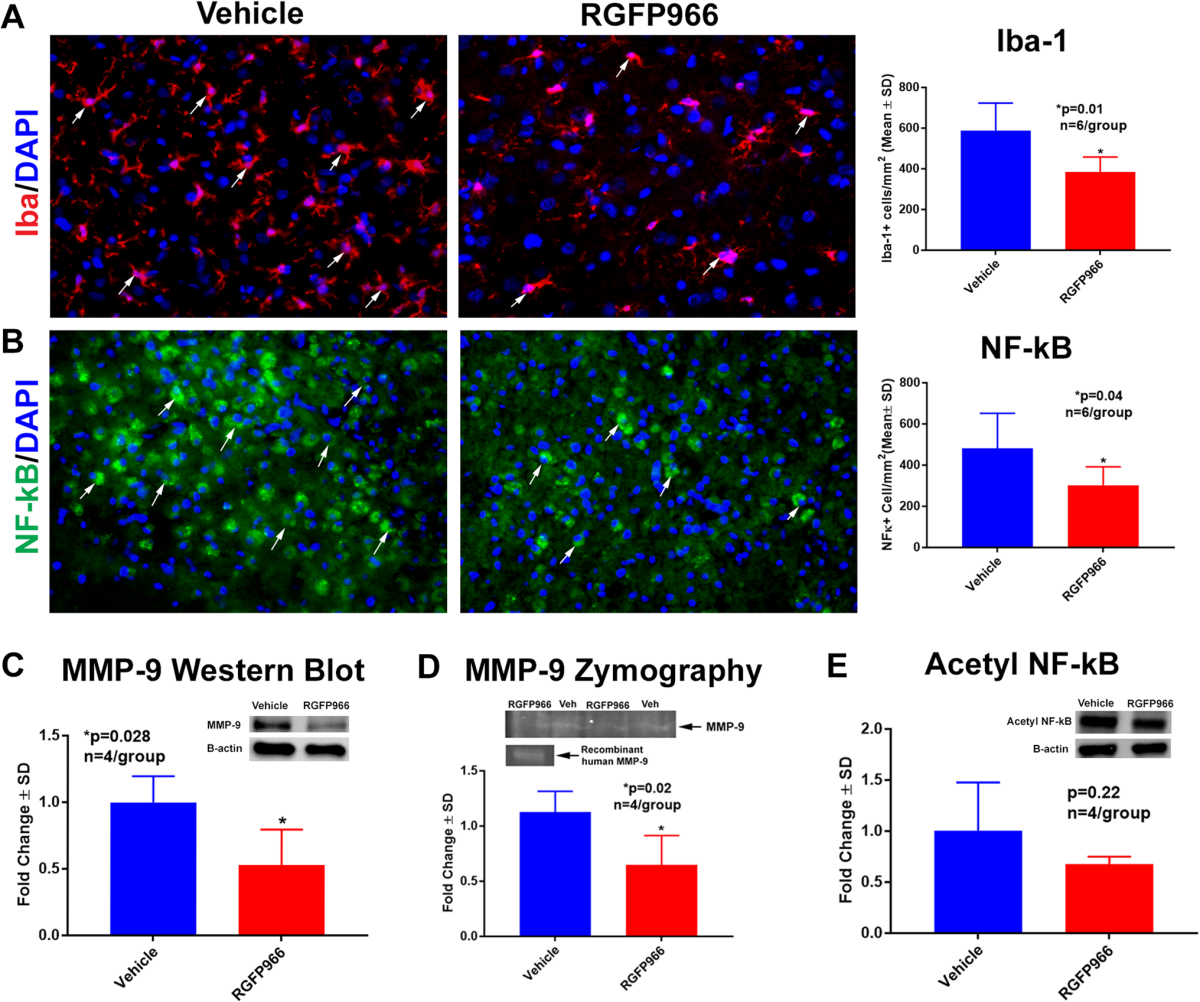
RGFP966 decreases inflammatory markers in the ischemic brain. **A**, **B** RGFP966 decreases the immunoreactivity of Iba-1 (**A**) and NF-kB (**B**) in the peri-infarct cortex. **C**, **D** RGFP966 decreases the protein level of MMP-9 as measured by Western Blot and the activity of MMP-9 as measured by MMP-9 zymography (**D**). There was no difference in the levels of acetyl NF-kB between the groups (**E**). SD, standard deviation

To further investigate whether RGFP966 treatment regulates neuroinflammation after experimental stroke, we measured activated microglia (Iba-1) and NF-kB in the peri-infarct cortex using immunostaining. As shown in Fig. 4A, B, selective inhibition of HDAC3 significantly decreased the immunoreactivity of Iba-1 and NF-kB–positive cells. Using Western blot, we showed that MMP9 protein levels were decreased in the ischemic brain of the RGFP966 group (Fig. 4C), and using MMP-9 zymography, we showed that the activity of MMP-9 is also significantly decreased in the ischemic brain of the treatment group (Fig. 4D). Western blot showed that RGFP966 did not alter acetylated NF-kB (Fig. 4E).

## Discussion

In the present study, we showed that selective inhibition of HDAC3 with RGFP966 decreases cerebral edema and BBB leakage in vivo and improves functional outcomes. The BBB protection mediated by RGFP966 is associated with increased tight junction proteins and decreased AQP-4 and neuroinflammation. These findings suggest that HDAC3 inhibition represents a promising new therapeutic option to target the underlying mechanisms of cerebral edema after stroke and slow its progression.

Cerebral edema is a devastating complication of stroke [5]. BBB dysfunction after ischemia contributes to cerebral edema [6, 65]. The BBB is a specialized multicellular structure that selectively separates the brain interstitium from the contents of the blood vessels [66, 67]. The BBB is a dynamic barrier, with active communication between cells of the BBB and the brain parenchymal cells [6]. The innermost layer of the BBB is comprised of endothelial cells and the BBB regulates the paracellular permeability of endothelial cells through junction proteins including tight junction (TJ) proteins [68]. Degradation of TJ proteins, such as claudin-5 and ZO-1, leads to increased permeability of BBB [8]. Emerging evidence suggests that epigenetic regulation plays an important role in BBB injury and recovery after stroke [13, 69]. Nonspecific HDAC inhibitors (e.g., valproic acid) have been shown to reverse the downregulation of ZO-1 and claudin-5 after MCAO [18]. Using in vitro model of ischemia, Zhao et al. showed that pretreatment with RGFP966 attenuated transendothelial cell permeability and upregulated claudin-5 [70]. In line with these reports, our data showed that selective inhibition of HDAC3 in vivo increases ZO-1 and claudin-5 in the ischemic brain.

Aquaporin channels have been shown to mediate the plasmalemmal water flux in cerebral edema [6]. In the brain, AQP4 is mainly expressed in the astrocytes [61] and it has been shown to facilitate astrocytic swelling [62–64]. Overexpression of AQP4 enhances ionic edema formation [62], and the deletion of AQP4 has been shown to impair cell water uptake in several brain injury models [63, 64]. Our data showed that selective inhibition of HDAC3 decreased the total AQP4 as well as the levels of AQP4 in GFAP-positive cells. We also found a significant correlation between HDAC3 and AQP4 in GFAP-positive cells, and the decrease of HDAC3 in GFAP-positive cells was correlated with better neurological scores and decreased cerebral edema. This data support the role of histone deacetylases such as HDAC3 in epigenetic regulation of AQP4 and cerebral edema after ischemic stroke.

Due to BBB breakdown after ischemic stroke, leukocytes infiltrate the brain tissue and cause inflammatory response resulting in secondary BBB disruption and worsening edema [71, 72]. Activated microglia outnumber the peripheral blood monocytes infiltrating the ischemic brain [73]; therefore, resident microglia are the main damaging inflammatory cells to BBB [7, 10]. MMP-9 is mainly derived from neutrophils and has been shown to play an important role in BBB disruption [74]. NF-kB is a central regulator of the inflammatory response [12] and it has been proposed that modulating the activity of NF-kB could attenuate inflammation after ischemic stroke [12, 75, 76].

Emerging evidence indicates that activation of immune cells after injury requires transcriptional changes that are highly regulated by epigenetic mechanisms [77, 78]. HDAC3 has been implicated in the regulation of inflammatory gene expression [79] and it has been shown to function as a brake of the alternative/anti-inflammatory activation [80]. Our data support that selective inhibition of HDAC3 regulates neuroinflammation after experimental stroke. We found that RGFP966 significantly decreases activated microglia and NF-kB in the ischemic brain. RGFP966 treatment also decreases the MMP-9.

The current study provides evidence that early administration of a selective HDAC3 inhibitor (RGFP966) in vivo decreases cerebral edema and BBB leakage after ischemic stroke. The BBB protection by RGFP966 is mediated in part by higher levels of tight junction proteins, downregulation of AQP4 and HDAC3 in astrocytes, as well as the attenuation of neuroinflammation. These data suggest that targeting HDAC3 might be a novel therapeutic approach for the treatment of cerebral edema after stroke.

## Supplementary Information

Figure 1S Full unedited western blot. Panel A-B shows full unedited western blot for MMP-9 (**A**) and Acetyl NF-KB (**B**) (PNG 461 kb)

High Resolution Image (TIF 3280 kb)

## Data Availability

The datasets generated during and/or analyzed during the current study are available from the corresponding author on reasonable request.

## References

[1] Virani SS, Alonso A, Benjamin EJ, Bittencourt MS, Callaway CW, Carson AP, Chamberlain AM, Chang AR, Cheng S, Delling FN (2020). Heart disease and stroke statistics-2020 update: a report from the American Heart Association. Circulation.

[2] Dirnagl U, Iadecola C, Moskowitz MA (1999). Pathobiology of ischaemic stroke: an integrated view. Trends Neurosci.

[3] Huttner HB, Schwab S (2009). Malignant middle cerebral artery infarction: clinical characteristics, treatment strategies, and future perspectives. Lancet Neurol.

[4] Powers WJ, Rabinstein AA, Ackerson T, Adeoye OM, Bambakidis NC, Becker K, Biller J, Brown M, Demaerschalk BM, Hoh B (2019). Guidelines for the early management of patients with acute ischemic stroke: 2019 update to the 2018 guidelines for the early management of acute ischemic stroke: a guideline for healthcare professionals from the American Heart Association/American Stroke Association. Stroke.

[5] Liebeskind DS, Juttler E, Shapovalov Y, Yegin A, Landen J, Jauch EC (2019). Cerebral edema associated with large hemispheric infarction. Stroke.

[6] Stokum JA, Gerzanich V, Simard JM (2016). Molecular pathophysiology of cerebral edema. J Cereb Blood Flow Metab.

[7] Chen S, Shao L, Ma L (2021). Cerebral edema formation after stroke: emphasis on blood-brain barrier and the lymphatic drainage system of the brain. Front Cell Neurosci.

[8] Wolburg H, Lippoldt A (2002). Tight junctions of the blood-brain barrier: development, composition and regulation. Vascul Pharmacol.

[9] Zador Z, Stiver S, Wang V, Manley GT (2009). Role of aquaporin-4 in cerebral edema and stroke. Handb Exp Pharmacol.

[10] Jolivel V, Bicker F, Biname F, Ploen R, Keller S, Gollan R, Jurek B, Birkenstock J, Poisa-Beiro L, Bruttger J (2015). Perivascular microglia promote blood vessel disintegration in the ischemic penumbra. Acta Neuropathol.

[11] Wang G, Guo Q, Hossain M, Fazio V, Zeynalov E, Janigro D, Mayberg MR, Namura S (2009). Bone marrow-derived cells are the major source of MMP-9 contributing to blood-brain barrier dysfunction and infarct formation after ischemic stroke in mice. Brain Res.

[12] Nurmi A, Lindsberg PJ, Koistinaho M, Zhang W, Juettler E, Karjalainen-Lindsberg ML, Weih F, Frank N, Schwaninger M, Koistinaho J (2004). Nuclear factor-kappaB contributes to infarction after permanent focal ischemia. Stroke.

[13] Stamatovic SM, Phillips CM, Martinez-Revollar G, Keep RF, Andjelkovic AV (2019). Involvement of epigenetic mechanisms and non-coding RNAs in blood-brain barrier and neurovascular unit injury and recovery after stroke. Front Neurosci.

[14] Jenuwein T, Allis CD (2001). Translating the histone code. Science.

[15] Kouzarides T (2007). Chromatin modifications and their function. Cell.

[16] Kim HJ, Rowe M, Ren M, Hong JS, Chen PS, Chuang DM (2007). Histone deacetylase inhibitors exhibit anti-inflammatory and neuroprotective effects in a rat permanent ischemic model of stroke: multiple mechanisms of action. J Pharmacol Exp Ther.

[17] Ren M, Leng Y, Jeong M, Leeds PR, Chuang DM (2004). Valproic acid reduces brain damage induced by transient focal cerebral ischemia in rats: potential roles of histone deacetylase inhibition and heat shock protein induction. J Neurochem.

[18] Wang Z, Leng Y, Tsai LK, Leeds P, Chuang DM (2011). Valproic acid attenuates blood-brain barrier disruption in a rat model of transient focal cerebral ischemia: the roles of HDAC and MMP-9 inhibition. J Cereb Blood Flow Metab.

[19] Kassis H, Chopp M, Liu XS, Shehadah A, Roberts C, Zhang ZG (2014). Histone deacetylase expression in white matter oligodendrocytes after stroke. Neurochem Int.

[20] Kim HJ, Leeds P, Chuang DM (2009). The HDAC inhibitor, sodium butyrate, stimulates neurogenesis in the ischemic brain. J Neurochem.

[21] Faraco G, Pancani T, Formentini L, Mascagni P, Fossati G, Leoni F, Moroni F, Chiarugi A (2006). Pharmacological inhibition of histone deacetylases by suberoylanilide hydroxamic acid specifically alters gene expression and reduces ischemic injury in the mouse brain. Mol Pharmacol.

[22] Mahlknecht U, Hoelzer D, Bucala R, Verdin E (1999). Cloning and characterization of the murine histone deacetylase (HDAC3**)**. Biochem Biophys Res Commun.

[23] Broide RS, Redwine JM, Aftahi N, Young W, Bloom FE, Winrow CJ (2007). Distribution of histone deacetylases 1–11 in the rat brain. J Mol Neurosci.

[24] Baltan S, Bachleda A, Morrison RS, Murphy SP (2011). Expression of histone deacetylases in cellular compartments of the mouse brain and the effects of ischemia. Transl Stroke Res.

[25] Matheson R, Chida K, Lu H, Clendaniel V, Fisher M, Thomas A, Lo EH, Selim M, Shehadah A (2020). Neuroprotective effects of selective inhibition of histone deacetylase 3 in experimental stroke. Transl Stroke Res.

[26] Yang X, Wu Q, Zhang L, Feng L (2016). Inhibition of histone deacetylase 3 (HDAC3) mediates ischemic preconditioning and protects cortical neurons against ischemia in rats. Front Mol Neurosci.

[27] Liao Y, Cheng J, Kong X, Li S, Li X, Zhang M, Zhang H, Yang T, Dong Y, Li J (2020). HDAC3 inhibition ameliorates ischemia/reperfusion-induced brain injury by regulating the microglial cGAS-STING pathway. Theranostics.

[28] Xia M, Zhao Q, Zhang H, Chen Y, Yuan Z, Xu Y, Zhang M (2017). Proteomic analysis of HDAC3 selective inhibitor in the regulation of inflammatory response of primary microglia. Neural Plast.

[29] Zhang L, He X, Liu L, Jiang M, Zhao C, Wang H, He D, Zheng T, Zhou X, Hassan A (2016). Hdac3 interaction with p300 histone acetyltransferase regulates the oligodendrocyte and astrocyte lineage fate switch. Dev Cell.

[30] Chen YT, Zang XF, Pan J, Zhu XL, Chen F, Chen ZB, Xu Y (2012). Expression patterns of histone deacetylases in experimental stroke and potential targets for neuroprotection. Clin Exp Pharmacol Physiol.

[31] Baltan S (2012). Histone deacetylase inhibitors preserve function in aging axons. J Neurochem.

[32] Zhao Q, Zhang F, Yu Z, Guo S, Liu N, Jiang Y, Lo EH, Xu Y, Wang X (2019). HDAC3 inhibition prevents blood-brain barrier permeability through Nrf2 activation in type 2 diabetes male mice. J Neuroinflammation.

[33] Zhao B, Yuan Q, Hou JB, Xia ZY, Zhan LY, Li M, Jiang M, Gao WW, Liu L (2019). Inhibition of HDAC3 ameliorates cerebral ischemia reperfusion injury in diabetic mice in vivo and in vitro. J Diabetes Res.

[34] Zhou X, Qiao B (2022). Inhibition of HDAC3 and ATXN3 by miR-25 prevents neuronal loss and ameliorates neurological recovery in cerebral stroke experimental rats. J Physiol Biochem.

[35] Zhang MJ, Zhao QC, Xia MX, Chen J, Chen YT, Cao X, Liu Y, Yuan ZQ, Wang XY, Xu Y (2020). The HDAC3 inhibitor RGFP966 ameliorated ischemic brain damage by downregulating the AIM2 inflammasome. FASEB J.

[36] Chou CJ, Herman D, Gottesfeld JM (2008). Pimelic diphenylamide 106 is a slow, tight-binding inhibitor of class I histone deacetylases. J Biol Chem.

[37] Xu C, Soragni E, Chou CJ, Herman D, Plasterer HL, Rusche JR, Gottesfeld JM (2009). Chemical probes identify a role for histone deacetylase 3 in Friedreich's ataxia gene silencing. Chem Biol.

[38] Rai M, Soragni E, Chou CJ, Barnes G, Jones S, Rusche JR, Gottesfeld JM, Pandolfo M (2010). Two new pimelic diphenylamide HDAC inhibitors induce sustained frataxin upregulation in cells from Friedreich's ataxia patients and in a mouse model. PLoS ONE.

[39] Malvaez M, McQuown SC, Rogge GA, Astarabadi M, Jacques V, Carreiro S, Rusche JR, Wood MA (2013). HDAC3-selective inhibitor enhances extinction of cocaine-seeking behavior in a persistent manner. Proc Natl Acad Sci U S A.

[40] Shehadah A, Chen J, Kramer B, Zacharek A, Cui Y, Roberts C, Lu M, Chopp M (2013). Efficacy of single and multiple injections of human umbilical tissue-derived cells following experimental stroke in rats. PLoS ONE.

[41] Chen J, Sanberg PR, Li Y, Wang L, Lu M, Willing AE, Sanchez-Ramos J, Chopp M (2001). Intravenous administration of human umbilical cord blood reduces behavioral deficits after stroke in rats. Stroke.

[42] Chen J, Li Y, Wang L, Zhang Z, Lu D, Lu M, Chopp M (2001). Therapeutic benefit of intravenous administration of bone marrow stromal cells after cerebral ischemia in rats. Stroke.

[43] Fisher M, Feuerstein G, Howells DW, Hurn PD, Kent TA, Savitz SI, Lo EH, Group S (2009). Update of the stroke therapy academic industry roundtable preclinical recommendations. Stroke.

[44] Swanson RA, Morton MT, Tsao-Wu G, Savalos RA, Davidson C, Sharp FR (1990). A semiautomated method for measuring brain infarct volume. J Cereb Blood Flow Metab.

[45] Barone FC, Clark RK, Feuerstein G, Lenkinski RE, Sarkar SK (1991). Quantitative comparison of magnetic resonance imaging (MRI) and histologic analyses of focal ischemic damage in the rat. Brain Res Bull.

[46] Fernandez-Lopez D, Faustino J, Daneman R, Zhou L, Lee SY, Derugin N, Wendland MF, Vexler ZS (2012). Blood-brain barrier permeability is increased after acute adult stroke but not neonatal stroke in the rat. J Neurosci.

[47] Wang C, Huang R, Li C, Lu M, Emanuele M, Zhang ZG, Chopp M, Zhang L (2019). Vepoloxamer enhances fibrinolysis of tPA (tissue-type plasminogen activator) on acute ischemic stroke. Stroke.

[48] Natarajan R, Northrop N, Yamamoto B (2017). Fluorescein isothiocyanate (FITC)-dextran extravasation as a measure of blood-brain barrier permeability. Curr Protoc Neurosci.

[49] Yao X, Derugin N, Manley GT, Verkman AS (2015). Reduced brain edema and infarct volume in aquaporin-4 deficient mice after transient focal cerebral ischemia. Neurosci Lett.

[50] Asahi M, Asahi K, Jung JC, del Zoppo GJ, Fini ME, Lo EH (2000). Role for matrix metalloproteinase 9 after focal cerebral ischemia: effects of gene knockout and enzyme inhibition with BB-94. J Cereb Blood Flow Metab.

[51] Frankowski H, Gu YH, Heo JH, Milner R, Del Zoppo GJ (2012). Use of gel zymography to examine matrix metalloproteinase (gelatinase) expression in brain tissue or in primary glial cultures. Methods Mol Biol.

[52] Toth M, Sohail A, Fridman R (2012). Assessment of gelatinases (MMP-2 and MMP-9) by gelatin zymography. Methods Mol Biol.

[53] Stroke Therapy Academic Industry R (1999). Recommendations for standards regarding preclinical neuroprotective and restorative drug development. Stroke.

[54] Patabendige A, Singh A, Jenkins S, Sen J, Chen R (2021). Astrocyte activation in neurovascular damage and repair following ischaemic stroke. Int J Mol Sci.

[55] Jukkola P, Gu C (2015). Regulation of neurovascular coupling in autoimmunity to water and ion channels. Autoimmun Rev.

[56] Sofroniew MV (2015). Astrocyte barriers to neurotoxic inflammation. Nat Rev Neurosci.

[57] Janzer RC, Raff MC (1987). Astrocytes induce blood-brain barrier properties in endothelial cells. Nature.

[58] Mathiisen TM, Lehre KP, Danbolt NC, Ottersen OP (2010). The perivascular astroglial sheath provides a complete covering of the brain microvessels: an electron microscopic 3D reconstruction. Glia.

[59] Attwell D, Buchan AM, Charpak S, Lauritzen M, Macvicar BA, Newman EA (2010). Glial and neuronal control of brain blood flow. Nature.

[60] Kitchen P, Salman MM, Halsey AM, Clarke-Bland C, MacDonald JA, Ishida H, Vogel HJ, Almutiri S, Logan A, Kreida S (2020). Targeting aquaporin-4 subcellular localization to treat central nervous system edema. Cell.

[61] Nielsen S, Nagelhus EA, Amiry-Moghaddam M, Bourque C, Agre P, Ottersen OP (1997). Specialized membrane domains for water transport in glial cells: high-resolution immunogold cytochemistry of aquaporin-4 in rat brain. J Neurosci.

[62] Yang B, Zador Z, Verkman AS (2008). Glial cell aquaporin-4 overexpression in transgenic mice accelerates cytotoxic brain swelling. J Biol Chem.

[63] Manley GT, Fujimura M, Ma T, Noshita N, Filiz F, Bollen AW, Chan P, Verkman AS (2000). Aquaporin-4 deletion in mice reduces brain edema after acute water intoxication and ischemic stroke. Nat Med.

[64] Papadopoulos MC, Manley GT, Krishna S, Verkman AS (2004). Aquaporin-4 facilitates reabsorption of excess fluid in vasogenic brain edema. FASEB J.

[65] Prakash R, Carmichael ST (2015). Blood-brain barrier breakdown and neovascularization processes after stroke and traumatic brain injury. Curr Opin Neurol.

[66] Haley MJ, Lawrence CB (2017). The blood-brain barrier after stroke: structural studies and the role of transcytotic vesicles. J Cereb Blood Flow Metab.

[67] Bernardo-Castro S, Sousa JA, Bras A, Cecilia C, Rodrigues B, Almendra L, Machado C, Santo G, Silva F, Ferreira L (2020). Pathophysiology of blood-brain barrier permeability throughout the different stages of ischemic stroke and its implication on hemorrhagic transformation and recovery. Front Neurol.

[68] Paris L, Tonutti L, Vannini C, Bazzoni G (2008). Structural organization of the tight junctions. Biochim Biophys Acta.

[69] Ihezie SA, Mathew IE, McBride DW, Dienel A, Blackburn SL, Thankamani Pandit PK (2021). Epigenetics in blood-brain barrier disruption. Fluids Barriers CNS.

[70] Zhao Q, Yu Z, Zhang F, Huang L, Xing C, Liu N, Xu Y, Wang X (2019). HDAC3 inhibition prevents oxygen glucose deprivation/reoxygenation-induced transendothelial permeability by elevating PPARgamma activity in vitro. J Neurochem.

[71] Enzmann G, Mysiorek C, Gorina R, Cheng YJ, Ghavampour S, Hannocks MJ, Prinz V, Dirnagl U, Endres M, Prinz M (2013). The neurovascular unit as a selective barrier to polymorphonuclear granulocyte (PMN) infiltration into the brain after ischemic injury. Acta Neuropathol.

[72] Sifat AE, Vaidya B, Abbruscato TJ (2017). Blood-brain barrier protection as a therapeutic strategy for acute ischemic stroke. AAPS J.

[73] Denes A, Vidyasagar R, Feng J, Narvainen J, McColl BW, Kauppinen RA, Allan SM (2007). Proliferating resident microglia after focal cerebral ischaemia in mice. J Cereb Blood Flow Metab.

[74] Gidday JM, Gasche YG, Copin JC, Shah AR, Perez RS, Shapiro SD, Chan PH, Park TS (2005). Leukocyte-derived matrix metalloproteinase-9 mediates blood-brain barrier breakdown and is proinflammatory after transient focal cerebral ischemia. Am J Physiol Heart Circ Physiol.

[75] Harari OA, Liao JK (2010). NF-kappaB and innate immunity in ischemic stroke. Ann N Y Acad Sci.

[76] Ali A, Shah FA, Zeb A, Malik I, Alvi AM, Alkury LT, Rashid S, Hussain I, Ullah N, Khan AU (2020). NF-kappaB inhibitors attenuate MCAO induced neurodegeneration and oxidative stress-a reprofiling approach. Front Mol Neurosci.

[77] Busslinger M, Tarakhovsky A (2014). Epigenetic control of immunity. Cold Spring Harb Perspect Biol.

[78] Phan AT, Goldrath AW, Glass CK (2017). Metabolic and epigenetic coordination of T cell and macrophage immunity. Immunity.

[79] Chen X, Barozzi I, Termanini A, Prosperini E, Recchiuti A, Dalli J, Mietton F, Matteoli G, Hiebert S, Natoli G (2012). Requirement for the histone deacetylase Hdac3 for the inflammatory gene expression program in macrophages. Proc Natl Acad Sci U S A.

[80] Mullican SE, Gaddis CA, Alenghat T, Nair MG, Giacomin PR, Everett LJ, Feng D, Steger DJ, Schug J, Artis D (2011). Histone deacetylase 3 is an epigenomic brake in macrophage alternative activation. Genes Dev.

