# Supplementary figures and images for: Histone Deacetylase 3 Inhibition Decreases Cerebral Edema and Protects the Blood–Brain Barrier After Stroke
Source: Mol Neurobiol. 2022 Oct 18;60(1):235–46. doi: 10.1007/s12035-022-03083-z (PMC9758108; doi:10.1007/s12035-022-03083-z)

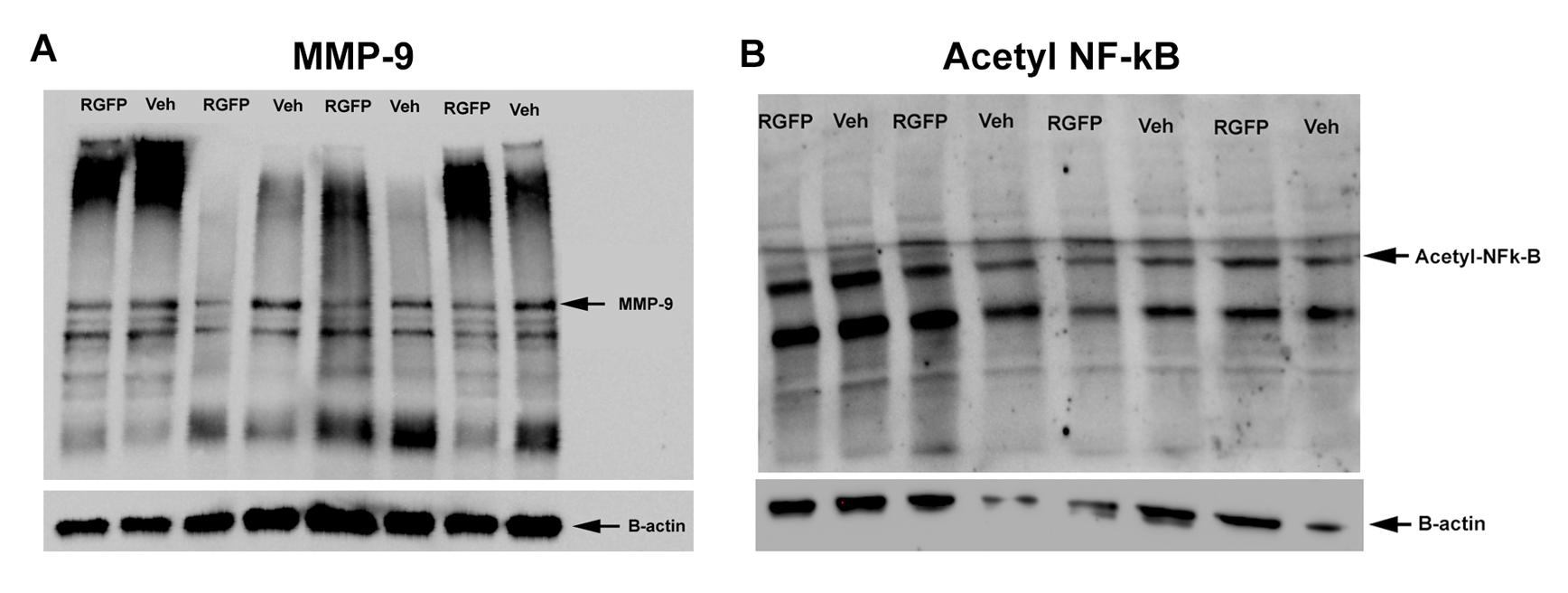

Supplement: Supplementary file 1 — Full unedited western blot. Panel A-B shows full unedited western blot for MMP-9 (A) and Acetyl NF-KB (B) (PNG 461 kb) [file 12035_2022_3083_Fig5_ESM.png]

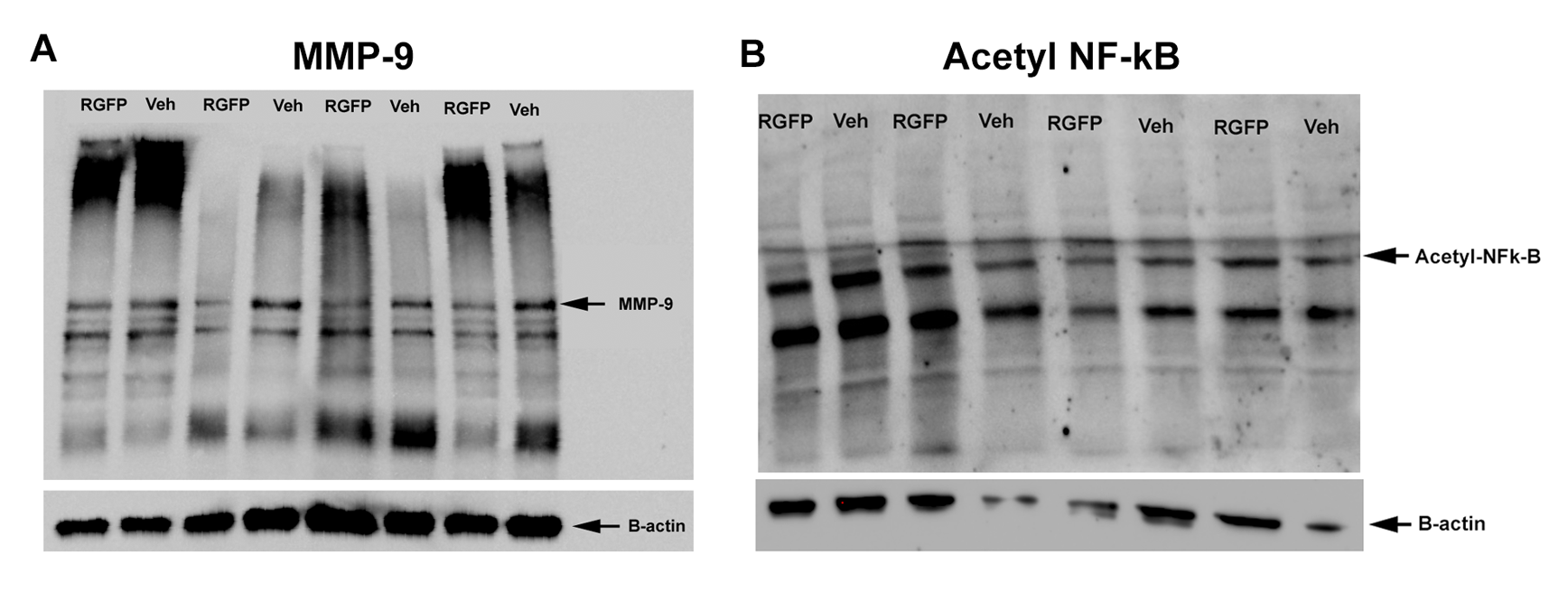

Supplement: Supplementary file 2 — High Resolution Image (TIF 3280 kb) [file 12035_2022_3083_MOESM1_ESM.tif]
